# Prognostic analysis of esophageal cancer patients after neoadjuvant therapy

**DOI:** 10.3389/fimmu.2025.1553086

**Published:** 2025-02-21

**Authors:** Jing Dong, Cheng Li, Bingxiang Wang, Yang Li, Suzhen Wang, Hongxia Cui, Min Gao

**Affiliations:** ^1^ Department of Oncology, Affiliated Tangshan Gongren Hospital, North China University of Science and Technology, Tangshan, China; ^2^ Quality Management Department, Shandong Cancer Hospital and Institute, Shandong First Medical University and Shandong Academy of Medical Sciences, Jinan, China; ^3^ Security Department Shandong Cancer Hospital and Institute, Shandong First Medical University and Shandong Academy of Medical Sciences, Jinan, China; ^4^ Department of Radiotherapy, Shandong Cancer Hospital and Institute, Shandong First Medical University and Shandong Academy of Medical Sciences, Jinan, China; ^5^ Department of Oncology, Jining First People’s Hospital, Jining, China

**Keywords:** esophageal cancer, neoadjuvant therapy, immunotherapy, chemotherapy, radiotherapy, prognosis

## Abstract

**Background:**

Neoadjuvant therapy is widely used for esophageal cancer (EC), but optimal treatment regimens and predictive factors for outcomes remain unclear. This study retrospectively analyzed data from EC patients who underwent neoadjuvant therapy.

**Methods:**

The *chi-square test* or *Fisher’s exact test* was utilized to examine differences in general clinicopathological data between treatment benefit groups. Survival analyses were conducted using Kaplan-Meier methods. Cox univariate and multivariate regression analyses were employed to identify independent risk factors affecting overall survival (OS) in EC patients receiving different treatment modalities.

**Results:**

The study included 175 EC patients who underwent neoadjuvant therapy. Analysis of clinical benefit differences revealed that patients aged < 65 years (*P* = 0.028) and those with esophageal squamous cell carcinoma (ESCC) (*P* = 0.027) were more likely to achieve a complete response, while N1 patients more frequently attained an objective response (*P* < 0.001). OS analysis indicated that patients who did not receive immunotherapy exhibited better survival outcomes compared to those who did (*P* = 0.002). Patients with pretreatment N3 status demonstrated poorer survival compared to those with N0 (*P* = 0.004), N1 (*P* = 0.003), and N2 (*P* = 0.003) status. Among post-neoadjuvant EC patients who did not receive immunotherapy, those with primary tumors located in the middle esophagus (*hazard ratio* [*HR*], 0.181; *95% Confidence interval* (*CI*) = 0.044-0.739; *P* = 0.017) and lower esophagus (*HR*, 0.163; *95%CI* = 0.032-0.821; *P* = 0.028) demonstrated a better prognosis compared to patients with tumors in the upper esophagus. Notably, EC patients who did not receive immunotherapy after neoadjuvant therapy and underwent 3-6 cycles of therapy exhibited a poorer prognosis compared to those who received 1-2 cycles (*HR*, 2.731; *95%CI* = 1.187-6.284; *P* = 0.018).

**Conclusions:**

In conclusion, this study found that immunotherapy did not play a decisive role in neoadjuvant EC therapy. Instead, 1-2 cycles of chemotherapy or chemoradiotherapy were associated with a more favorable prognosis for these patients.

## 1 Introduction

Esophageal cancer (EC) is a malignant neoplasm of the gastrointestinal tract characterized by high morbidity and mortality rates. The etiology of EC is multifaceted and potentially linked to various factors, including poor dietary habits and genetic predisposition. EC exhibits an insidious onset, with minimal symptomatic manifestation in early stages, and is predominantly diagnosed in advanced stages, resulting in an overall poor prognosis with a 5-year survival rate of 15-25% (1, 2). Currently, surgical intervention remains the primary treatment modality for early-stage EC. The majority of patients eligible for direct surgery undergo comprehensive treatment, principally centered on surgical intervention (3). However, patients with locally advanced EC exhibit low R0 resection rates, frequently experience high recurrence and metastasis rates following surgical treatment alone, and do not achieve high 5-year survival rates (4). With advancements in surgical techniques and the incorporation of neoadjuvant therapies, the prognosis for patients with locally advanced EC has significantly improved. Relevant clinical studies have substantiated the safety and efficacy of neoadjuvant radiotherapy and neoadjuvant chemotherapy (5).

For locally advanced EC, neoadjuvant chemotherapy and neoadjuvant chemoradiotherapy are common treatment options that can effectively enhance prognosis, efficacy, and confer survival benefits to patients (6). Furthermore, advancements in targeted and immunological drugs have provided new perspectives on neoadjuvant therapy selection (7). Current neoadjuvant treatment strategies encompass chemotherapy, radiotherapy, molecular-targeted therapy, immunotherapy, and other integrated approaches. However, the optimal selection among these options remains a subject of debate and necessitates further investigation.

Recent immunotherapy studies have demonstrated that the combination of chemotherapy and immunotherapy is more effective than chemotherapy alone in the first-line treatment of advanced EC (8). However, the efficacy and safety of immunotherapy combined with chemotherapy for neoadjuvant treatment of locally advanced EC remain controversial. While research has shown that immunotherapy significantly improves the 5-year survival rate in patients with advanced EC, there is limited data on neoadjuvant therapy for resectable locally advanced EC. This study aims to investigate the impact of neoadjuvant therapy, with or without immunotherapy, on the prognosis of patients with Stage II or III EC (T2-T4, N0-N+), providing a reference for clinical treatment decisions.

## 2 Materials and methods

### 2.1 Subjects of the study

Patients with EC undergoing neoadjuvant therapy at Shandong Cancer Hospital between January 2021 and December 2023 were enrolled in this study. The inclusion criteria were as follows: (1) age ≥ 18 years; (2) EC patients with American Joint Committee on Cancer 8th edition staging of Stage II or III (T2-T4, N0-N+) confirmed by histopathologic and imaging tests; (3) Eastern Cooperative Oncology Group performance status score of 0-2; (4) no contraindication to chemotherapy and receipt of at least one cycle of platinum-based agent with paclitaxel chemotherapy (21 days apart between each period); and (5) if neoadjuvant radiotherapy was received, the radiotherapy regimen was required to be Dt: 41.4 Gy, 1.8 Gy * 23f, 1.8 Gy/f, 5f/w. The exclusion criteria were (1) complicated severe organ diseases; (2) incomplete clinical data; and (3) unacceptable treatment toxicities or treatment delays. This study was approved by the ethics committee of Shandong Cancer Hospital (approval number: SDTHEC202412018). Informed consent was obtained from all patients.

### 2.2 General information

A retrospective study methodology was employed to gather patients’ demographic and clinical data, including age, sex, BMI, smoking and drinking status, primary tumor location, pathological type, chemotherapy cycles, treatment modality, initial clinical staging, and postoperative pathological staging. Follow-up assessments were conducted quarterly for the first two years post-treatment, then biannually thereafter, to determine survival status and time to fatal event, with follow-up extending to September 1, 2024. Overall survival (OS) was defined as the duration from diagnosis to death from any cause or the date of the last follow-up visit. Clinical benefits were categorized according to the Response Evaluation Criteria in Solid Tumors (RECIST) guidelines, encompassing complete response (CR), partial response (PR), stable disease (SD), and progressive disease (PD).

### 2.3 Statistical analysis

For data analysis, R 4.2.2 software was utilized. Count data were presented as frequency and percentage (n, %). Differences in the general clinicopathological data among different treatment benefit groups were evaluated using the *chi-square test* or *Fisher’s exact test*. Kaplan-Meier analyses were employed for survival assessments. To analyze independent risk factors affecting postoperative survival of patients with EC treated with various modalities, univariate and multivariate Cox regression analyses were conducted. Statistical significance was set at *P* < 0.05.

## 3 Results

### 3.1 Baseline characteristics

A cohort of 175 patients with EC who had undergone previous neoadjuvant therapy was analyzed. The majority of patients were under 65 years old (100, 57.1%) and male (150, 85.7%). Treatment modalities included chemotherapy (36, 20.6%), immunotherapy combined with chemotherapy (89, 50.9%), chemoradiotherapy (34, 19.4%), and immunotherapy combined with chemoradiotherapy (16, 9.1%). Following neoadjuvant therapy, clinical benefit was assessed: 64 (36.6%) patients achieved CR, 70 (40%) PR, 33 (18.9%) SD, and 8 (4.6%) PD. These outcomes were determined based on pathologic analyses of tumor specimens post-surgery. CR was defined as the complete disappearance of all target lesions with no new lesions for at least 3 months. PR was characterized by a ≥ 30% decrease in the sum of target lesion diameters. PD was identified by the emergence of new lesions or at least a 20% increase in the sum of target lesion diameters. SD was classified as a reduction in the sum of the maximum diameter of target lesions that did not meet PR criteria or an increase that did not meet PD criteria. **Table 1** presents the baseline characteristics of the patients.

**Table 1:** Demographic, clinical and pathologic features of patients.

Characteristic	Case (%)	Characteristic	Case (%)
**Age**		**Clinical T-stages** ^a^	
< 65 years	100 (57.1%)	cT2	14 (8%)
≥ 65 years	75 (42.9%)	cT3	154 (88%)
**Gender**		cT4	7 (4%)
Female	25 (14.3%)	**Clinical N-stages** ^a^	
Male	150 (85.7%)	cN0	47 (26.9%)
**BMI**		cN1	95 (54.3%)
< 25	134 (76.6%)	cN2	29 (16.6%)
≥ 25	41 (23.4%)	cN3	4 (2.3%)
**Smoking**		**Clinical M-stages** ^a^	
Never smoking	87 (49.7%)	cM0	175 (100%)
Former/Current smoking	88 (50.3%)	cM1	0 (0%)
**Drinking**		**Pathological T-stage** ^a^	
Never drinking	92 (52.6%)	pT0	78 (44.6%)
Former/Current drinking	83 (47.4%)	pT1	22 (12.6%)
**Tumor Location** ^a^		pT2	22 (12.6%)
The upper esophagus	18 (10.3%)	pT3	44 (25.1%)
The middle esophagus	94 (53.7%)	pT4	9 (5.1%)
The lower esophagus	63 (36%)	**Pathological N-stage** ^a^	
**Pathological types** ^b^		pN0	127 (72.6%)
EAC	9 (5.1%)	pN1	26 (14.9%)
ESCC	166 (94.9%)	pN2	17 (9.7%)
**Number of chemotherapy cycles**		pN3	5 (2.9%)
1-2	121 (69.1%)	**Pathological M-stage** ^a^	
3-6	54 (30.9%)	pM0	174 (99.4%)
**Treatment Modality** ^c^		pM1	1 (0.6%)
C	36 (20.6%)	**Clinical benefit** ^d^	
IC	89 (50.9%)	CR	64 (36.6%)
CR	34 (19.4%)	PR	70 (40%)
ICR	16 (9.1%)	SD	33 (18.9%)
		PD	8 (4.6%)

^a^Lesion location and tumor staging were classified according to the 8th edition of the AJCC classification; ^b^EAC, esophageal adenocarcinoma; ESCC, esophageal squamous cell carcinoma; ^c^C, chemotherapy; CR, chemotherapy combined with radiotherapy; IC, immune checkpoint inhibitors combined with chemotherapy; ICR, immune checkpoint inhibitors combined with chemotherapy and radiotherapy; ^d^CR, complete response; OR, objective response.

### 3.2 Analysis of differences in different clinical benefits

In this study, OR was defined as CR plus PR. As presented in **Table 2**, among the 175 patients included in this study, 64 (36.6%) achieved CR and 134 (76.6%) achieved OR. Notably, CR was attained more frequently in patients younger than 65 years (*P* = 0.028) and in those diagnosed with esophageal squamous cell carcinoma (ESCC) (*P* = 0.027). The clinical N-stage demonstrated a statistically significant influence on OR achievement, with a higher proportion of N1 patients achieving OR (*P* < 0.001).

**Table 2:** The differences in clinical benefit among patients with diverse clinical characteristics.

Characteristic	CR ^a^			OR ^b^		
	Non-CR (n = 111)	Achieving CR (n = 64)	*P*	Non-OR (n = 41)	Achieving OR (n = 134)	*P*
Age			0.028			0.075
< 65 years	56 (50.5%)	44 (68.8%)		18 (43.9%)	82 (61.2%)	
≥ 65 years	55 (49.5%)	20 (31.3%)		23 (56.1)	52 (38.8%)	
Gender			0.132			0.856
Female	12 (10.8%)	13 (20.3%)		5 (12.2%)	20 (14.9%)	
Male	99 (89.2%)	51 (79.7%)		36 (87.8%)	114 (85.1%)	
BMI				0.095			0.641
< 25	90 (81.1%)	44 (68.8%)		33 (80.5%)	101 (75.4%)	
≥ 25	21 (18.9%)	20 (31.3%)		8 (19.5%)	33 (24.6%)	
Smoking			0.4			0.967
Never smoking	52 (46.8%)	35 (54.7%)		21 (51.2%)	66 (49.3%)	
Former/Current smoking	59 (53.2%)	29 (45.3%)		20 (48.8%)	68 (50.7%)	
Drinking			> 0.99			0.463
Never drinking	58 (52.3%)	34 (53.1%)		19 (46.3%)	73 (54.5%)	
Former/Current drinking	53 (47.7%)	30 (46.9%)		22 (53.7%)	61 (45.5%)	
Tumor location ^c^			0.359			0.214
The upper esophagus	9 (8.1%)	9 (14.1%)		2 (4.9%)	16 (11.9%)	
The middle esophagus	59 (53.2%)	35 (54.7%)		20 (48.8%)	74 (55.2%)	
The lower esophagus	43 (38.7%)	20 (31.3%)		19 (46.3%)	44 (32.8%)	
Pathological type ^d^			0.027			0.034
EAC	9 (8.1%)	0 (0%)		5 (12.2%)	4 (3%)	
ESCC	102 (91.9%)	64 (100%)		36 (87.8%)	130 (97%)	
Number of chemotherapy cycle			0.202			> 0.99
1-2	81 (73%)	40 (62.5%)		28 (68.3%)	93 (69.4%)	
3-6	30 (27%)	24 (37.5%)		13 (31.7%)	41 (30.6%)	
Treatment modality ^e^			0.117			0.689
C + CR	39 (35.1%)	31 (48.4%)		18 (43.9%)	52 (38.8%)	
IC + ICR	72 (64.9%)	33 (51.6%)		23 (56.1%)	82 (61.2%)	
Clinical T-stage ^c^			0.074			0.05
cT2	5 (4.5%)	9 (14.1%)		0 (0%)	14 (10.4%)	
cT3	101 (91%)	53 (82.8%)		40 (97.6%)	114 (85.1%)	
cT4	5 (4.5%)	2 (3.1%)		1 (2.4%)	6 (4.5%)	
Clinical N-stage ^c^			0.575			0.001
cN0	31 (27.9%)	16 (25%)		18 (43.9%)	29 (21.6%)	
cN1	56 (50.5%)	39 (60.9%)		12 (29.3%)	83 (61.9%)	
cN2	21 (18.9%)	8 (12.5%)		9 (22%)	20 (14.9%)	
cN3	3 (2.7%)	1 (1.6%)		2 (4.9%)	2 (1.5%)	

^a^CR, complete response; ^b^OR, objective response; ^c^The lesion location and tumor staging were classified according to the 8th edition of the AJCC classification; ^d^EAC, esophageal adenocarcinoma; ESCC, esophageal squamous cell carcinoma; ^e^C, chemotherapy; CR, chemotherapy combined with radiotherapy; IC, immune checkpoint inhibitors combined with chemotherapy; ICR, immune checkpoint inhibitors combined with chemotherapy and radiotherapy.

Regarding therapeutic factors, 40 (62.5%) of the 64 patients who achieved CR received 1-2 cycles of neoadjuvant therapy. Additionally, 93 (69.4%) of the 134 patients who achieved OR received 1-2 cycles of neoadjuvant therapy. Among the 121 patients who received 1-2 cycles of treatment, 40 (33.1%) achieved CR and 93 (76.9%) achieved OR. Of the 54 patients who received 3-6 cycles of treatment, 24 (44.4%) achieved CR and 41 (75.9%) achieved OR. The number of neoadjuvant cycles did not demonstrate statistically significant differences in the achievement of CR or OR (*P >*0.05). Furthermore, 31 (48.4%) of the 64 patients who achieved CR did not receive immunotherapy during their neoadjuvant therapy regimen. Of the 134 patients who achieved OR, 52 (38.8%) did not receive immunotherapy during their neoadjuvant therapy regimen. In the group without combination immunotherapy (70 patients), 31 (44.3%) achieved CR and 52 (74.3%) achieved OR. Among the 105 patients in the combination immunotherapy group, 33 (31.4%) achieved CR and 82 (78.1%) achieved OR. The study found no statistically significant differences in the effects of combination immunotherapy on achieving CR and OR (*P* > 0.05).

### 3.3 Survival analysis

**Figure 1:**
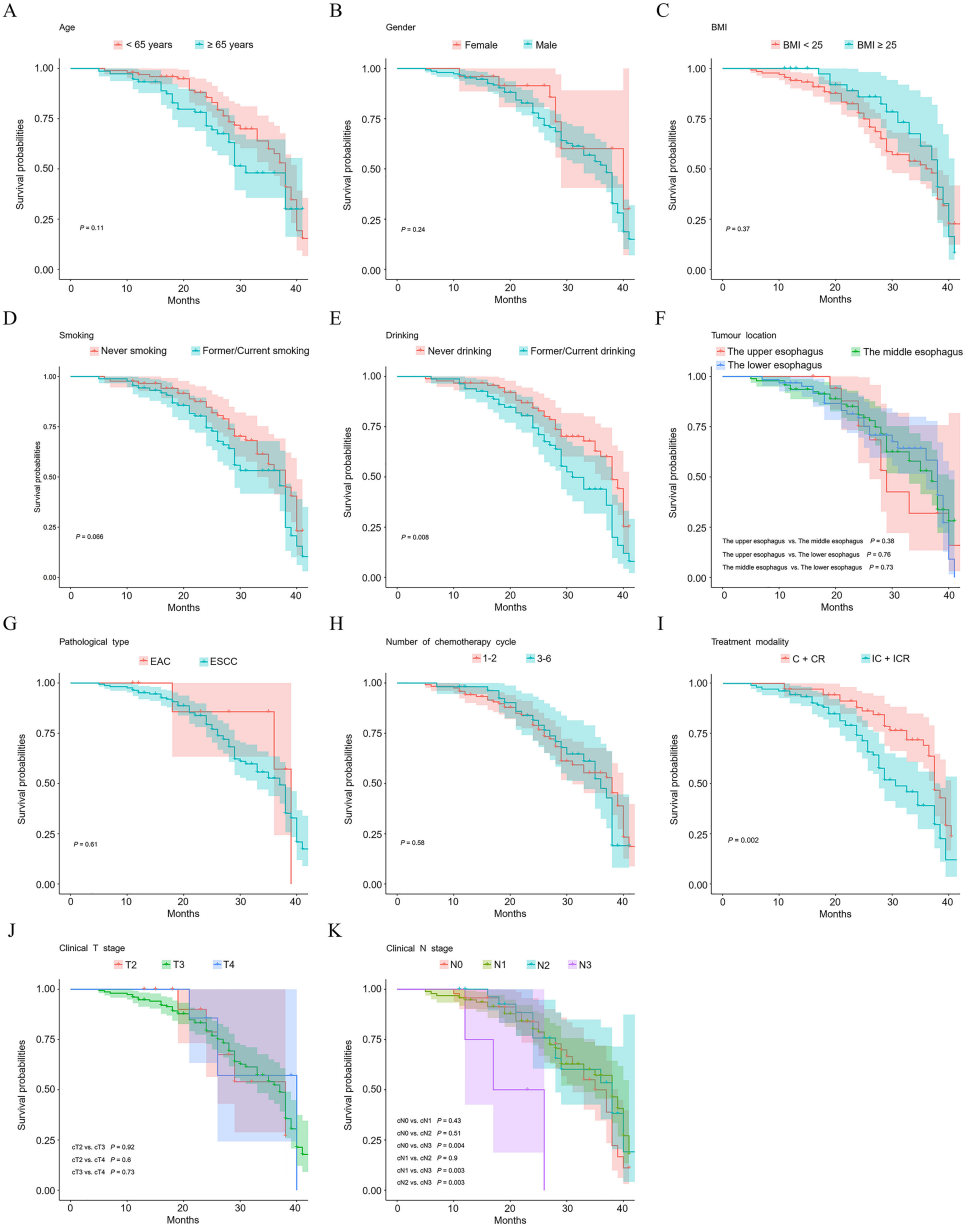
Kaplan–Meier plots of overall survival probability of esophageal cancer patients. Kaplan-Meier survival curves illustrate survivals of patients with different age **(A)**, gender **(B)**, BMI **(C)**, smoking status **(D)**, drinking status **(E)**, tumour location **(F)**, pathological type **(G)**, number of chemotherapy cycle **(H)**, treatment modality **(I)**, clinical T stage **(J)**, and clinical N stage **(K)**.

Survival analysis was conducted using the Kaplan-Meier method. As illustrated in **Figure 1E**, patients who abstained from alcohol consumption demonstrated improved OS compared to those with a history of or current alcohol use (*P* = 0.008). Interestingly, patients who did not undergo immunotherapy as part of their treatment regimen exhibited better survival rates than those who did (*P* = 0.002) (**Figure 1I**). **Figure 1K** reveals that regarding pretreatment N-staging, N3 patients had significantly poorer survival outcomes compared to N0 (*P* = 0.004), N1 (*P* = 0.003), and N2 (*P* = 0.003) patients. No statistically significant differences in OS were observed among patients with other varying general clinicopathological factors (*P* > 0.05).

**Figure 2:**
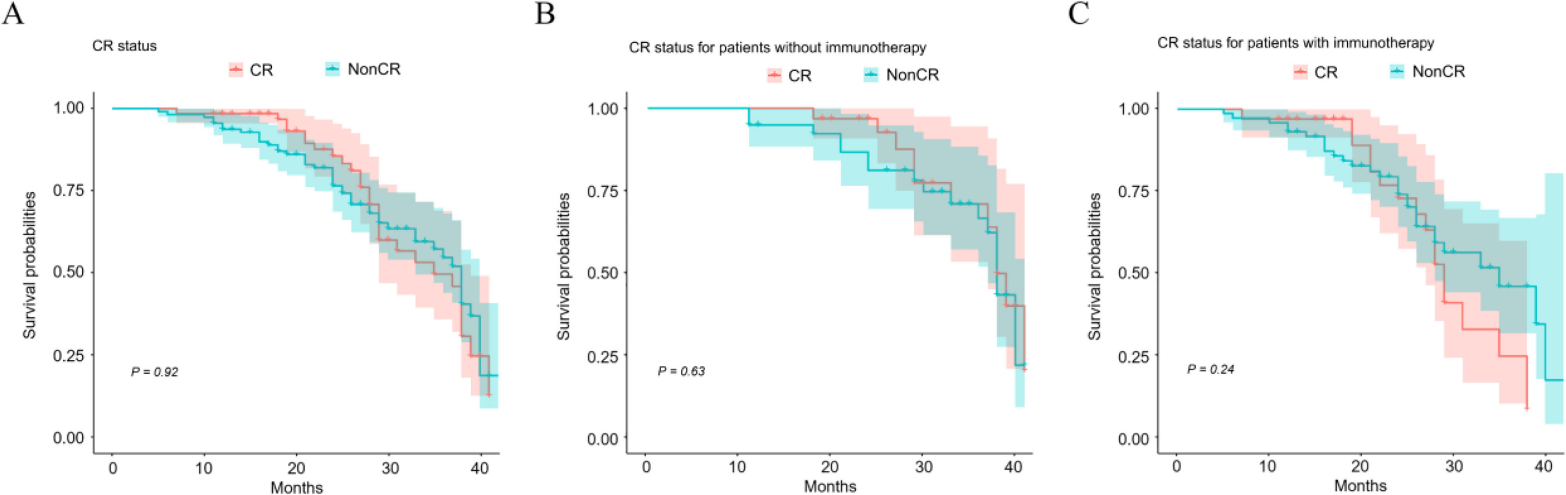
Impact of CR status on overall patient survival after neoadjuvant therapy. Kaplan-Meier survival curves illustrate survivals of patients with different CR status **(A)**, CR status for patients without **(B)** or with immunotherapy **(C)**.

To elucidate the influence of CR on overall survival in EC patients who underwent neoadjuvant therapy, statistical analyses were conducted. The findings indicated that CR following neoadjuvant therapy did not significantly affect the overall survival of the 175 patients studied (*P* = 0.92), as illustrated in **Figure 2A**. Furthermore, no substantial survival differences were observed in either subgroup of patients: those who did not received immunotherapy therapy as part of their neoadjuvant regimen (**Figure 2B**, *P* = 0.63) and those who did (**Figure 2C**, *P* = 0.24).

### 3.4 Univariate and multivariate Cox analyses

**Figure 3:**
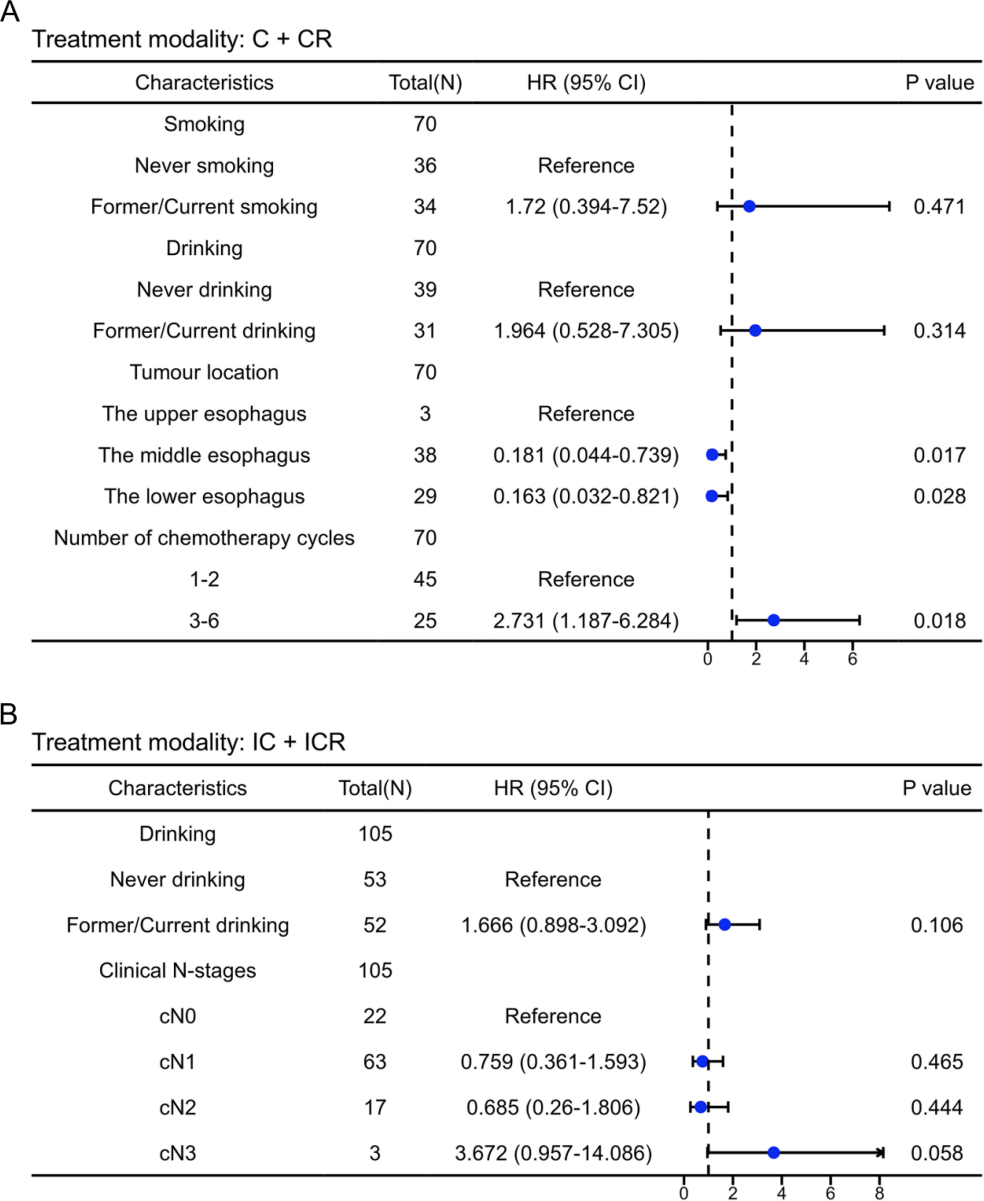
Multivariate cox regression analysis of the factors associated with overall survival of esophageal cancer patients. Forest plots from multivariate Cox regression analysis of neoadjuvant regimens in EC without **(A)** or with **(B)** combined immunotherapy.

In this study, univariate and multivariate Cox analyses were conducted to identify independent prognostic factors in patients receiving different treatment modalities. **Table 3** presents the results of the univariate Cox analysis. Factors with *P* < 0.15 were included in the multivariate Cox analysis, and the results were visualized as forest plots. **Figure 3A** illustrates that among patients with EC after neoadjuvant therapy who did not receive immunotherapy, those with primary tumors located in the middle (*HR*, 0.181; *95%CI* = 0.044-0.739; *P* = 0.017) and lower esophagus (*HR*, 0.163; *95%CI* = 0.032-0.821; *P* = 0.028) had a better prognosis compared to patients with tumors in the upper esophagus. Importantly, among EC patients who received neoadjuvant therapy without combination immunotherapy, those who underwent 3-6 cycles of therapy demonstrated a worse prognosis than those who received 1-2 cycles of neoadjuvant therapy (*HR*, 2.731; *95%CI* = 1.187-6.284; *P* = 0.018). This finding suggests that for patients receiving neoadjuvant chemotherapy or chemoradiotherapy, increasing the number of treatment cycles did not confer a survival benefit. Instead, it led to a worse prognosis. Furthermore, in EC patients who received neoadjuvant therapy containing immunotherapy, no significant effect of alcohol consumption and T-staging on patient prognosis was observed (**Figure 3B**).

**Table 3:** Univariate Cox regression analysis of factors influencing patient prognosis across different treatment groups.

Characteristic	C + CR ^a^ (n = 70)		IC + ICR ^a^ (n = 105)	
	*HR* (*95%CI*)	*P*	*HR* (*95%CI*)	*P*
Age				
< 65 years	Reference		Reference	
≥ 65 years	1.413 (0.671-2.975)	0.363	1.334 (0.729-2.442)	0.35
Gender				
Female	Reference		Reference	
Male	1.341 (0.467-3.851)	0.586	1.725 (0.616-4.833)	0.299
BMI				
< 25	Reference		Reference	
≥ 25	0.777 (0.316-1.91)	0.582	1.413 (0.711-2.809)	0.324
Smoking				
Never smoking	Reference		Reference	
Former/Current smoking	1.889 (0.906-3.94)	0.09	1.47 (0.809-2.674)	0.206
Drinking				
Never drinking	Reference		Reference	
Former/Current drinking	2.063 (0.998-4.265)	0.051	1.778 (0.981-3.223)	0.058
Tumor location ^b^				
The upper esophagus	Reference		Reference	
The middle esophagus	0.393 (0.112-1.382)	0.146	1.205 (0.517-2.806)	0.666
The lower esophagus	0.579 (0.164-2.047)	0.396	1.157 (0.461-2.902)	0.756
Pathological types ^c^				
ECA	Reference		Reference	
ESCC	0.863 (0.203-3.674)	0.842	1.766 (0.236-13.22)	0.58
Number of chemotherapy cycles				
1-2	Reference		Reference	
3-6	1.855 (0.874-3.936)	0.107	0.908 (0.473-1.741)	0.77
Clinical stages ^b^				
cT2	Reference		Reference	
cT3	9285115 (0-Inf)	0.997	1.087 (0.427-2.771)	0.861
cT4	9111510 (0-Inf)	0.997	0.82 (0.094-7.156)	0.857
Clinical N-stages ^b^				
cN0	Reference		Reference	
cN1	0.594 (0.269-1.313)	0.198	0.775 (0.369-1.629)	0.502
cN2	0.748 (0.267-2.097)	0.581	0.751 (0.289-1.947)	0.555
cN3	2.219e-07 (0-Inf)	0.998	4.825 (1.298-17.926)	0.019

^a^C, chemotherapy; CR, chemotherapy combined with radiotherapy; IC, immune checkpoint inhibitors combined with chemotherapy; ICR, immune checkpoint inhibitors combined with chemotherapy and radiotherapy; ^b^Lesion location and tumor staging were classified according to the 8th edition of the AJCC classification; ^c^EAC, esophageal adenocarcinoma; ESCC, esophageal squamous cell carcinoma.

## 4 Discussion

Patients with locally advanced EC often present with large tumors that are closely associated with surrounding tissues. In some cases, these tumors invade adjacent structures such as the aorta and thoracic duct, and may exhibit local lymph node metastasis. The complexity of these cases precludes direct surgical intervention, resulting in low R0 resection rates and poor long-term postoperative prognoses (9). In recent years, the widespread adoption of neoadjuvant therapy has significantly altered the treatment landscape for patients with locally advanced EC (10, 11). This study aims to further investigate the independent prognostic factors in EC patients following neoadjuvant therapy through retrospective analysis. The objective is to provide a reference basis for informed decision-making regarding EC neoadjuvant therapy regimens.

Multiple studies have confirmed that neoadjuvant chemoradiotherapy and neoadjuvant chemotherapy are more efficacious than surgery alone, without increasing the incidence of perioperative complications (12). As immunotherapy has gained widespread clinical application for advanced EC, researchers have begun exploring its combination with chemotherapy in neoadjuvant treatment to enhance EC prognosis (13). However, consensus on optimal EC neoadjuvant therapy remains elusive. Research indicates that preoperative immunotherapy can activate the immune system, enhance tumor-specific T cell activity through tumor antigens, and elicit therapeutic responses in primary tumors and metastatic lesions (14). Nonetheless, this study did not demonstrate a significant difference in clinical benefit or OS between neoadjuvant therapy regimens with or without immunotherapy.

Yang et al. conducted a study involving 16 patients with locally advanced ESCC who underwent neoadjuvant therapy. The treatment consisted of two cycles of carilizumab combined with a TC regimen of chemotherapy (paclitaxel plus carboplatin), followed by surgery 4 weeks after completion. Their findings indicated an objective remission rate of 81.3% and a CR rate of 31.3% following neoadjuvant therapy (15). Similarly, in the present study, 36.6% of patients achieved CR and 76.6% achieved OR after receiving neoadjuvant therapy. Research has shown that immunotherapy can suppress tumor angiogenesis and enhance the body’s anti-tumor response, while chemotherapy can amplify this effect (16). Furthermore, immunotherapy may augment the cytotoxic impact of chemotherapy on tumor cells by increasing chemosensitivity in patients with locally advanced EC (17). In this study, 44.3% of patients in the non-combination immunotherapy group and 31.4% in the combination immunotherapy group achieved CR. Although the CR rate was higher in the non-combination immunotherapy group, no statistically significant difference was observed in the effect of the combination immunotherapy regimen on achieving CR within the treatment modality. This outcome may be attributed to the fact that 28.6% of patients in this study received synchronized radiotherapy. Radiotherapy plays a crucial role in EC treatment and has consistently held a significant position in neoadjuvant therapy for EC (18, 19). Neoadjuvant chemotherapy and radiotherapy work synergistically, not only controlling local tumors but also addressing other hidden foci, thereby reducing the risk of disease recurrence and ultimately improving patient survival rates.

Neoadjuvant regimens for EC are widely utilized in clinical practice, with variations in treatment protocols and cycle numbers, including weekly regimens and 21-day cycles (20, 21). The number of neoadjuvant therapy cycles a patient receives depends on both the lesion’s response to treatment and the clinician’s decision-making process. In this study, 121 (69.1%) patients received 1-2 cycles of neoadjuvant chemotherapy, while 54 (30.9%) received 3-6 cycles. Multivariate Cox analysis results indicated that among EC patients who underwent neoadjuvant therapy without immunotherapy, those receiving 3-6 cycles had a poorer prognosis compared to those receiving 1-2 cycles. This suggests that for patients undergoing neoadjuvant chemotherapy or chemoradiotherapy, an increase in treatment cycles may negatively impact prognosis. However, this study did not find that the number of neoadjuvant therapy cycles affected the prognosis of EC patients receiving neoadjuvant therapy that included immunotherapy.

Recent years have witnessed continuous advancements in EC neoadjuvant therapy. Research has demonstrated that this approach not only effectively eliminates subclinical metastatic foci and reduces tumor stage, but also enhances surgical resection rates while minimizing the risk of tumor implantation and metastasis, thereby conferring survival benefits to EC patients (22, 23). Our findings indicate that following neoadjuvant therapy, a higher proportion of patients under 65 years of age or with ESCC pathology achieved CR. Notably, we observed that patients who did not receive immunotherapy in their treatment regimen exhibited better survival outcomes compared to those who did. Furthermore, among post-neoadjuvant EC patients not receiving immunotherapy, those undergoing 1-2 cycles of neoadjuvant therapy demonstrated a more favorable prognosis than those receiving 3-6 cycles. However, the present study has some limitations because it is a single-center retrospective analysis. Future prospective multicenter studies are required to corroborate our findings to further clarify the prognosis of different neoadjuvant treatment regimens for EC and the differences in survival among patients with different clinicopathological factors. Such investigations will facilitate more standardized and personalized treatment approaches, ultimately enhancing patients’ quality of survival.

## Data Availability

The raw data supporting the conclusions of this article will be made available by the authors, without undue reservation.
